# Investigation of the effects of an 8-week cross-country skiing exercise program on various reaction time parameters, selective attention and academic achievement in adolescents

**DOI:** 10.1186/s13102-024-00908-3

**Published:** 2024-05-24

**Authors:** Musab Çağın, Sezen Çimen Polat, Halil Sarol, Amador García Ramos, Abdulkerim Çeviker

**Affiliations:** 1 Department of Physical Education and Sports Teaching, Faculty of Sport Science, Gazi University, Ankara, Turkey; 2 Department of Coaching Education, Faculty of Sport Science, Gazi University, Ankara, Turkey; 3 Department of Recreation, Faculty of Sport Science, Gazi University, Ankara, Turkey; 4 Department of Physical Education and Sport, Faculty of Sport Sciences, University of Granada, Granada, Spain; 5 Department of Recreation, Faculty of Sport Science, Hitit University, Çorum, Turkey

**Keywords:** Ski, Reaction time, Academic achievement, Selective attention

## Abstract

Exercise slows or helps reverse the shrinkage of key cognitive brain regions such as the hippocampus, which is important for information processing, learning, reasoning and planning. For this reason, it is thought that regular exercise of individuals, especially during adolescence, which is considered one of the most important processes of development, can increase their performance in areas where cognitive activities are at the forefront. Cross-country skiing, one of the leading branches of winter sports, has a much more complex structure, unlike the branches that are widely preferred today (football, basketball, volleyball, etc.) and is a branch where many motor skills are exhibited at the same time For this reason, the effect of cross-country skiing, which is defined as more complex and difficult in terms of biomotor than other branches, on cognitive activities is a matter of curiosity. Therefore, the aim of the research is; to examine the cognitive effects of cross-country skiing exercise. The study involved 54 (26 male, 28 female) adolescents who had no prior experience in any licensed sports. The average age of the participants was determined as 12.61 ± 1.32. The participants were divided in experimental and control groups. Reaction performances were determined using the ÇAĞIN Hand and Foot Reaction Tests, selective attention performances were evaluated using the Flanker Test and academic achievement was determined using the e-Okul system. Two-way ANOVA revealed significant group × time interactions for hand and foot simple, selective, discriminative reaction time, selective attention and academic achievement (*p* < 0.05) due to improved values at post-test for the experimental group but not for the control groups. No significant group × time interactions were observed for correct reaction rate for all reaction parameters (*p* > 0.05). The study concludes that the cross-country skiing exercise, which was applied to adolescents for eight weeks, had a positive impact on the parameters of reaction, selective attention and academic achievement. Therefore, parents are suggested to encourage their children to exercise and engage in sports practices like cross-country skiing to improve cognitive and academic performance during adolescence.

## Introduction

Sports is an important tool for the physical, spiritual and social development of especially adolescent individuals. Research shows that individuals who start sports at an early age have better physical fitness than their peers, have a higher level of self-confidence, and are more aware of obeying the rules, respecting the rights of others, and communicating with people. Therefore, sports play a key role in raising a healthy generation [1–4]. If we examine the sports individually, it can be said that the skills required to be displayed in each branch have differed. For example, while explosive strength and endurance are at the forefront in athletics, it can be said that coordination and decision-making skills are at the forefront in archery. At the same time, it can be said that the more stimuli and variables an athlete is exposed to in a branch, the more skills the athlete must demonstrate at the same time. For this reason, performing complex branches in which more than one skill is at the forefront can bring a cognitive burden to the athletes. The athlete’s constant exposure to this cognitive load causes; It can lead to the development of decision-making, selective attention, cognitive flexibility and reaction skills [5]. For this reason, it is thought that directing individuals in adolescence to more complex branches may also contribute to their cognitive development.

If we classify the branches according to the stimuli and variables they contain, it can be said that winter sports have a more complex structure. In addition to the performance they must demonstrate, athletes must also cope with weather conditions, ground, altitude and difficulties specific to the winter season. Otherwise, the athlete may not be able to perform as desired, but may also face serious injuries [6]. For this reason, it can be said that doing winter sports involves having a serious level of attention. When we examine winter sports in terms of branches, it is observed that cross-country skiing is widely preferred.

Cross-country skiing is the origin of all skiing branches and is considered the predecessor of winter sports. It is a sport that uses all major muscle groups in a harmonious way. Cross-country skiing requires strength, endurance, coordination, balance, and flexibility. Athletes who can exhibit good skiing technique are able to use their energy more efficiently, giving them an advantage. In order to perform well in cross-country skiing, the athlete must have both technical and motoric skills at a certain level [7].

Neuroplasticity refers to the change in function and structure of neurons in the brain and synapses formed by these neurons due to certain environmental stimuli [8, 9]. Exercise is a process that activates cellular and molecular steps that support and help maintain brain plasticity [10]. Regular exercise has been found to facilitate the neuroplasticity of various brain structures and the hippocampus in both humans and animals. This supports cognitive functions and behavioral responses [11, 12]. Additionally, exercise can slow down or even reverse the shrinkage of basic cognitive brain regions such as the hippocampus that are important for information processing, learning, reasoning and planning [13]. Various studies have demonstrated that aerobic-based exercises increase cerebral blood volume and the number of hippocampus neurons [14, 15]. Furthermore, studies have shown that regular exercise has a positive impact on academic achievement, cognitive flexibility, and problem-solving skills [16–18]. This suggests that individuals who exercise regularly are likely to have better cognitive performance than those who do not exercise or lead a sedentary lifestyle.

When the studies examining the effects of exercise on cognitive activities are examined, it is observed that similar exercises are generally performed under the same environmental conditions. The fact that there have been no previous studies examining the effects of winter sports on cognitive activities makes the research even more important. Because cross-country skiing is very different from conventional branches in terms of the way it is practiced, the season, the weather conditions and the equipment used. Although the effects of winter sports on physical performance are known, more research is needed to determine their effects on cognitive performance.

In light of this information; It is thought that cross-country skiing, which includes instant decision-making and implementation, fast and accurate reaction to incoming stimuli, high level of attention to avoid obstacles, and being successful by displaying these components together, can improve reaction time, selective attention and decision-making skills in adolescent individuals. Therefore, the hypothesis of the research is that cross-country skiing exercise will positively affect cognitive activities in adolescent individuals. To investigate these effects, this study aims to examine the impact of an 8-week cross-country skiing exercise program on various reaction time parameters, selective attention and academic achievement in adolescents.

## Materials and method

### Participant

A total of 54 (26 male, 28 female) adolescents who had no previous licensed sports experience were included in the study. The participants were randomly divided into two even groups; experimental (26) and control (28). To be eligible for the study, participants had to meet the following criteria: no prior history of licensed sports, no cognitive impairment, no acute injury, and no school absenteeism. Experimental group had a mean age of 12.62 ± 1.16 years, mean height 153.85 ± 9.54 cm, and mean body mass 44.92 ± 7.49 kg. The control group had a mean age of 12.61 ± 1.47 years, mean height 155.07 ± 10.83 cm, and mean body mass 49.11 ± 7.74 kg.

### Study design

The necessary permissions and approvals were obtained from the Ethics Commission of Gazi University (Code: 2023-168) and the study was conducted in accordance with the Declaration of Helsinki. Participation in the study was voluntary, and the parents of the students signed a consent form, which informed them about all details of the study. The students were randomly divided into two groups; experimental and control groups. Both groups took reaction, selective attention, and academic achievement pretests. The tests were administered under similar conditions for both groups. The experimental group underwent eight weeks of cross-country skiing exercise while the control group continued their routine physical education classes. These exercises were applied to the experimental group during January and February. At the end of eight weeks, the post-tests were conducted for both groups, and the measurements of the study were completed. The study collected data on the age, body weight, and height of the participants through a personal information form. The reaction performance of the subjects was evaluated using the ÇAĞIN Hand and Foot Reaction Tests, while selective attention performance was measured through the Flanker Test. Additionally, the academic achievement of the participants was determined by using the E-Okul system.

### Exercise applications

The experimental group did cross-country skiing exercises twice a week (Tuesday and Friday) in addition to physical education for 8 weeks, while the control group attended only physical education classes twice a week. The exercises for the experimental group were conducted in December and January, at an altitude of 1838 m and a temperature range of -10 to -1 degrees Celsius. During the 8-week period, the skiing exercises began with a 10-min warm-up, followed by instruction on the proper technique according to the exercise program, application of the exercise, and ended with a 10-min cool-down period. As the weeks progressed, the time spent on technique instruction was gradually decreased, and the duration of the exercises was increased. By the 7th and 8th weeks, repetition exercises were performed, which included a combination of all the techniques taught over the previous six weeks (Table 1).

**Table 1:** The 8-week program of the experimental and control groups

**Experimental group**				**Control group**
**Week**	**Name of technique**	**Technique instruction time (min)**	**Exercise time (min)**	
1	Step	30	30	Participation in Physical Education Class 2 Days a Week
2	Double Pole	25	35	
3	One Step Double Pole	20	40	
4	Landing	15	45	
5	Herringbone Climbing	10	50	
6	Snow Slingshot	10	50	
7	Repetition of All Techniques	5	55	
8	Repetition of All Techniques	5	55	

### Data collection tools

#### ÇAĞIN hand and foot reaction tests

**Fig. 1:**
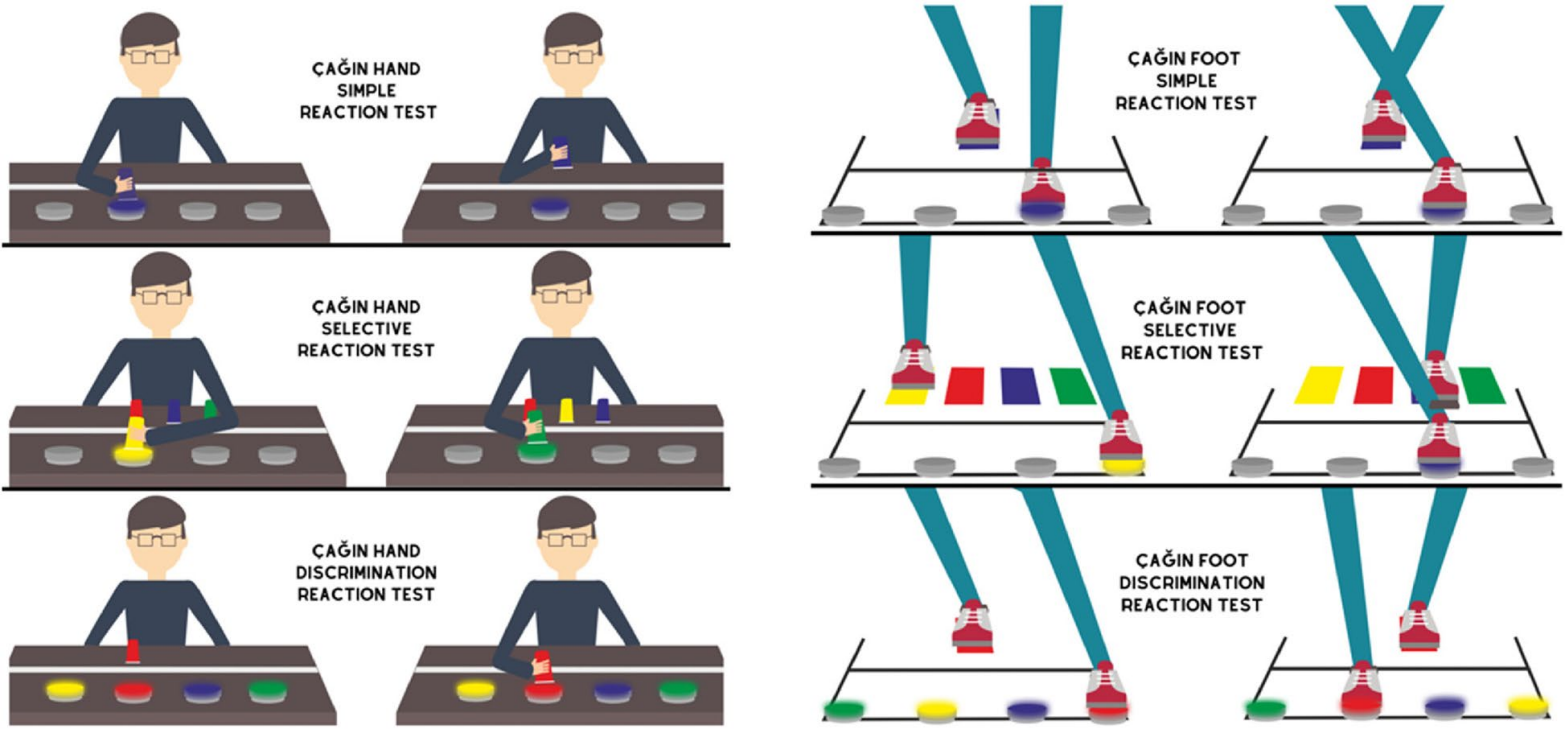
ÇAĞIN hand and foot reaction tests

The study measured the reaction performances of participants using the ÇAĞIN Hand and Foot Reaction Tests [19], which involved the use of a FitLight Trainer (CA/Ontario) device. Prior to conducting the reaction tests, individuals were assessed using the ÇAĞIN Color Blindness Test to ensure they met the inclusion criteria. The protocol involved administering simple, selective and discriminative reaction tests for hand and foot reactions to the participants (Fig. 1). Participants were asked to turn off the lights that were on for 20 s as quickly as possible, according to the specified reaction test. At the end of the test, the participants’ average reaction time and correct reaction rate were recorded. The test was repeated twice and the best scores were evaluated.

#### Flanker test

**Fig. 2:**
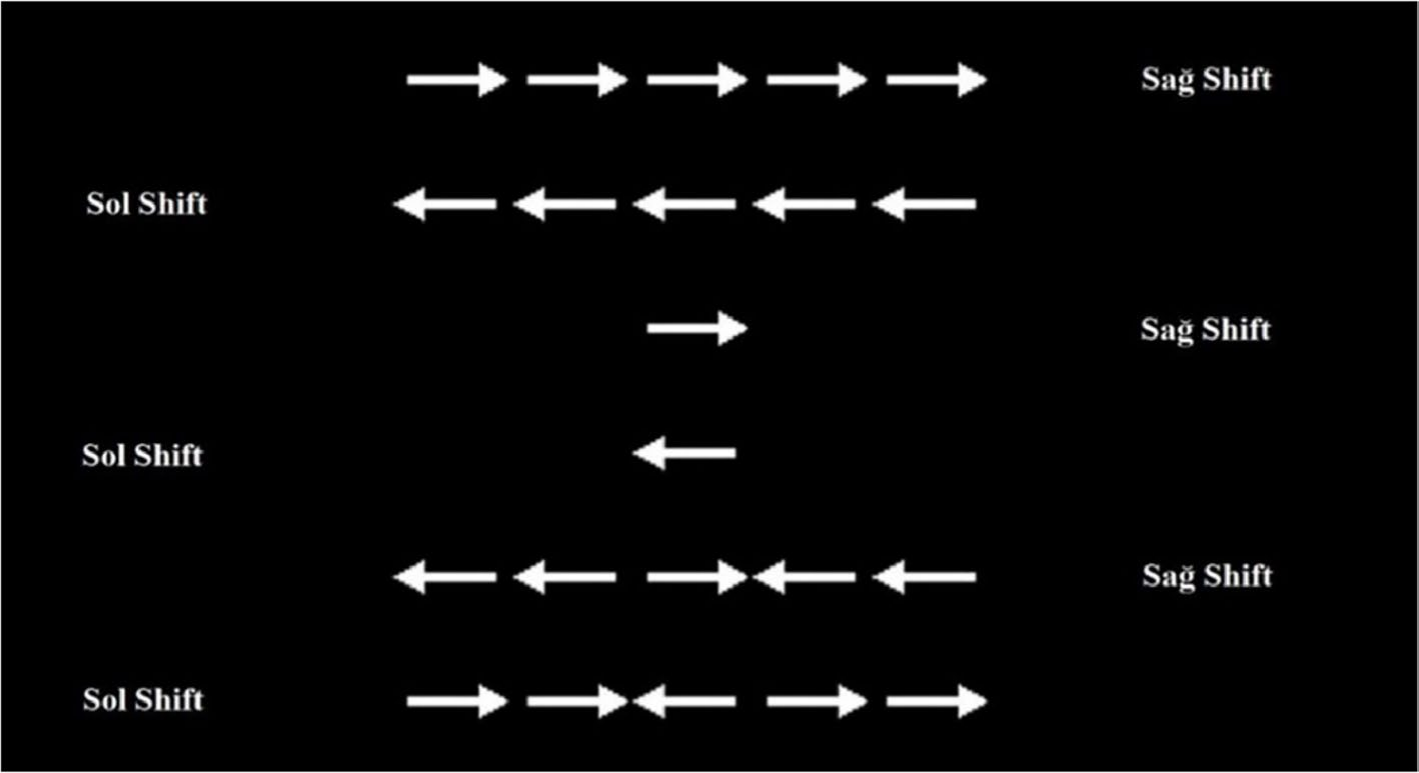
Descriptive visual representing the stimuli applied during the Flanker Test and the keyboard keys to be pressed by the subjects

The Flanker test is a type of psychophysical test measures selective attention performance. It was administered in a computerized environment using version 2.1 of the PEBL cognitive test battery [20]. During the test, arrows arranged horizontally side by side in groups of fives or singles appear in the center of the computer screen (Fig. 2). If the arrows appeared in singles, the subject was asked to press either the “Right Shift” or “Left Shift” key on the keyboard according to the direction indicated by the arrow. If the arrows were displayed in groups of five, the subject was asked to press either the “Right Shift” or “Left Shift” key on the keyboard according to the direction indicated by the arrow in the middle of the group. A total of 120 stimuli were given during the test, with each stimulus displayed on the screen for 500 ms. The stimulus was displayed on the screen for 500 ms following the subject’s response. A preliminary test consisting of 12 stimuli was administered prior to the actual test. The test lasted approximately five minutes. At the end of the test, the participants' total selective attention correct rate was recorded and evaluated.

#### Academic achievement

To evaluate the academic performance of the participants, the Ministry of National Education of the Republic of Turkey used the e-Okul system, which is a student grade tracking module. Participants' scores were graded according to the 100-point system. Higher scores mean that participants have better academic success. The study considered the semester grade point averages of the subjects before and after they participated in cross-country skiing exercise.

#### Data analysis

Analysis of the data obtained from the study was performed with the SPSS program (version 26.0; IBM Corp., Armonk, NY). In the study, mean and standard deviation values were taken as descriptive statistics. Two-way repeated measures analysis of variance (ANOVA) with group (control vs. experimental) as between-subject factor and time (pretest and posttest) as within subject factor were used to compare the different dependent variables. The confidence interval was chosen as 95% and values below *p* < 0.05 were considered statistically significant.

## Results

The interaction ANOVAs were significant for both hand and foot reaction parameters (*p* < 0.05). After the experiment, better improvements were detected in the experimental group compared to the control group (Table 2).

**Table 2:** Comparison of reaction times between the control and experimental groups

**Parameters**	**Variables**	**n**	**Pre test**	**Post test**	**Total**	**%Δ**	**F**	***p***
			$\overline{\rm X}$** ± SS**	$\overline{\rm X}$** ± SS**	$\overline{\rm X}$** ± SS**			
**Hand** **Simple** **R.T** **(sec)**	Experiment	26	.86 ± .12	.66 ± .07	.76 ± .02	%-23.26	4.841	.032
	Control	28	.80 ± .13	.84 ± .12	.82 ± .02	%5		
	Total	54	.83 ± .13	.75 ± .13				
	F = 33.314; *p* = .000					Group X Time Interaction F = 65.702; *p* = .000		
**Hand** **Selective** **R.T** **(sec)**	Experiment	26	1.46 ± .21	1.29 ± .18	1.37 ± .03	%-11.64	.008	.927
	Control	28	1.37 ± .22	1.37 ± .23	1.36 ± .03	%0		
	Total	54	1.41 ± .22	1.33 ± .21				
	F = 18.369; *p* = .000					Group X Time Interaction F = 16.086; *p* = .000		
**Hand** **Discrimination** **R.T** **(sec)**	Experiment	26	.86 ± .10	.71 ± .07	.79 ± .02	%-17.44	.126	.724
	Control	28	.79 ± .17	.81 ± .16	.80 ± .02	%2.53		
	Total	54	.83 ± .15	.76 ± .13				
	F = 46.721; *p* = .000					Group X Time Interaction F = 73.550; *p* = .000		
**Foot** **Simple** **R.T** **(sec)**	Experiment	26	1.02 ± .16	.82 ± .09	.92 ± .02	%-19.60	.119	.732
	Control	28	.95 ± .12	.91 ± .17	.93 ± .02	%-4.21		
	Total	54	.99 ± .15	.87 ± .14				
	F = 40.992; *p* = .000					Group X Time Interaction F = 17.509; *p* = .000		
**Foot** **Selective** **R.T** **(sec)**	Experiment	26	1.50 ± .16	1.34 ± .21	1.42 ± .05	%-10.66	.002	.966
	Control	28	1.40 ± .31	1.43 ± .36	1.42 ± .05	%2.14		
	Total	54	1.45 ± .25	1.39 ± .30				
	F = 6.798; *p* = .012					Group X Time Interaction F = 13.864; *p* = .000		
**Foot** **Discrimination** **R.T** **(sec)**	Experiment	26	.98 ± .09	.73 ± .09	.86 ± .02	%-25.51	4.094	.048
	Control	28	.93 ± .13	.90 ± .16	.92 ± .02	%-3.22		
	Total	54	.96 ± .12	.82 ± .15				
	F = 70.641; *p* = .000					Group X Time Interaction F = 43.637; *p* = .000		

*R.T*. Reaction Time, *sec* second, *%Δ* percentage differences

The confidence interval was chosen as 95% and values below *p* < 0.05 were considered statistically significant

The interaction ANOVAs were not significant for any reaction parameters (*p* > 0.05). After the experiment, similar results were detected in the experimental and control groups (Table 3).

**Table 3:** Comparison of reaction correct rate between the control and experimental groups

**Parameters**	**Variables**	**n**	**Pre test**	**Post test**	**Total**	**%Δ**	**F**	***p***
			$\overline{\rm X}$** ± SS**	$\overline{\rm X}$** ± SS**	$\overline{\rm X}$** ± SS**			
**Hand** **Simple** **R.C.R** **(%)**	Experiment	26	87.85 ± 5.60	89.50 ± 4.09	88.67 ± .93	%1.87	1.174	.284
	Control	28	87.00 ± 5.51	87.54 ± 4.71	87.26 ± .90	%0.62		
	Total	54	87.41 ± 5.52	88.48 ± 4.49				
	F = 6.307; *p* = .015					Group X Time Interaction F = 1.645; p = .205		
**Hand** **Selective** **R.C.R** **(%)**	Experiment	26	79.31 ± 5.39	81.00 ± 5,85	80.15 ± ,92	%2.13	.099	.754
	Control	28	80.07 ± 3.75	79.43 ± 5,64	79.75 ± ,88	%-0.79		
	Total	54	79.70 ± 4.58	80.19 ± 5,75				
	F = .734; *p* = .396					Group X Time Interaction F = 3.634; *p* = .062		
**Hand** **Discrimination** **R.C.R** **(%)**	Experiment	26	84.42 ± 5.70	84.27 ± 5.37	84.34 ± 1.12	%-0.17	.432	.514
	Control	28	83.39 ± 6.67	83.25 ± 5.77	83.32 ± 1.08	%-0.16		
	Total	54	83.89 ± 6.19	83.74 ± 5.55				
	F = .132; *p* = .718					Group X Time Interaction F = .000; *p* = .989		
**Foot** **Simple** **R.C.R** **(%)**	Experiment	26	86.65 ± 5.09	88.23 ± 5.87	87.44 ± 1.09	%1.82	5.173	.027
	Control	28	84.54 ± 7.05	83.43 ± 6.02	83.98 ± 1.05	%-1.31		
	Total	54	85.56 ± 6.22	85.74 ± 6.37				
	F = .131; *p* = .719					Group X Time Interaction F = 4.284; *p* = .043		
**Foot** **Selective** **R.C.R** **(%)**	Experiment	26	80.62 ± 4.30	83.31 ± 4.55	81.96 ± .91	%3.33	2.765	.102
	Control	28	78.96 ± 6.76	80.71 ± 5.33	79.83 ± .88	%2.21		
	Total	54	79.76 ± 5.72	81.96 ± 5.09				
	F = 9,820; *p* = ,003					Group X Time Interaction F = .442; *p* = .509		
**Foot** **Discrimination** **R.C.R** **(%)**	Experiment	26	83.23 ± 6.19	83.38 ± 6.13	82.93 ± .99	%0.18	.184	.670
	Control	28	82.64 ± 8.14	82.57 ± 6.93	82.97 ± .89	%-0.08		
	Total	54	82.93 ± 7.21	82.96 ± 6.51				
	F = .002; *p* = .965					Group X Time Interaction F = .014; *p* = .905		

*R.C.R.* Reaction Correct Rate, *%Δ* percentage differences

The confidence interval was chosen as 95% and values below *p* < 0.05 were considered statistically significant

The interaction of ANOVAs was significant for the selective attention parameter (*p* < 0.05). After the experiment, better improvements were detected in the experimental group compared to the control group (Table 4).

**Table 4:** Comparison of selective attention correct rate between the control and experimental groups

**Parameter**	**Variables**	**n**	**Pre test**	**Post test**	**Total**	**%Δ**	**F**	***p***
			$\overline{\rm X}$** ± SS**	$\overline{\rm X}$** ± SS**	$\overline{\rm X}$** ± SS**			
**Selective** **Attention** **C.R** **(%)**	Experiment	26	81.03 ± 19.25	90.96 ± 7.21	81.32 ± 2.21	%12.25	2.272	.138
	Control	28	81.60 ± 11.69	81.93 ± 7.35	86.44 ± .993	%0.40		
	Total	54	81.33 ± 15.64	86.28 ± 8.53				
	F = 7.87; *p* = .007					Group X Time Interaction F = 6.915; *p* = .011		

*C.R.* Correct Rate, *%Δ* percentage differences

The confidence interval was chosen as 95% and values below *p* < 0.05 were considered statistically significant

The interaction of ANOVAs was significant for the academic achievement parameter. After the experiment, better improvements were detected in the experimental group compared to the control group (Table 5).

**Table 5:** Comparison of participants’ academic achievement score averages according to groups and measurement times

**Parameter**	**Variables**	**n**	**Pre test**	**Post test**	**Total**	**%Δ**	**F**	***p***
			$\overline{\rm X}$** ± SS**	$\overline{\rm X}$** ± SS**	$\overline{\rm X}$** ± SS**			
**Academic** **Success** **(Point)**	Experiment	26	68.96 ± 10.96	74.31 ± 11.42	70.98 ± 1.42	%7.75	.282	.598
	Control	28	73.00 ± 9.94	72.96 ± 8.19	73.63 ± 1.34	%-0.05		
	Total	54	71.06 ± 10.54	73.61 ± 9.81				
	F = 5.722; *p* = .020					Group X Time Interaction F = 5.877; *p* = .019		

*%Δ* percentage differences

The confidence interval was chosen as 95% and values below *p* < 0.05 were considered statistically significant

## Discussion

This research was conducted on a total of 54 individuals divided in experimental (*n* = 26) and control (*n* = 28) groups. ANOVA interaction results show that the hand and foot simple, selective and discrimination reaction times of the experimental group are better than the control group. In this context, it is observed that cross-country exercise positively affects reaction time. There was no significant difference between the two groups in terms of correct reaction rate. After examining the literature, researchers discovered that implementing an exercise program for adolescent subjects over a 12-week period had a positive impact on their visual and auditory reaction time [21]. Additionally, it was observed that 8-10 year old male children who participated in judo technical training and games for 12 weeks showed improvement in their reaction performance [22]. Another study was conducted on sedentary individuals aged 18-25 years, which found that moderate intensity aerobic exercise can acutely shorten reaction time [23]. Therefore, it can be concluded that the findings of the present study are consistent with the existing literature on this subject.

ANOVA interaction results show that the selective attention performance of the experimental group is better than the control group. In this context, it is observed that cross-country skiing exercise positively affects selective attention. Research shows that short-term, high-intensity intermittent exercise is effective in developing selective attention among university students [24]. Another study found that acute aerobic exercise significantly improved selective attention in low-income children [25]. Additionally, selective attention and concentration levels increased as physical fitness levels increased in a study conducted on adolescents [26]. According to a study conducted on 14–15-year-old adolescents, it was observed that those who exercised regularly had a relatively better level of selective attention [27]. Therefore, it can be inferred that the study’s findings align with the existing literature on the topic.

ANOVA interaction results show that the academic achievement performance of the experimental group is better than the control group. In this context, it is observed that cross-country exercise positively affects academic success. According to Yıldız’s study, middle school male students who engaged in physical activity regularly had better academic achievement [28]. However, İşgüder’s study on high school students showed that academic achievement decreased as physical activity and sports participation increased [29]. According to a study involving university students, their academic performance was found to be positively correlated with their level of physical activity [30]. Another study also found that regular engagement in sports activities had a positive impact on academic achievement [31].

When the research results are evaluated holistically; It appears that cross-country skiing exercise positively affects all cognitive activities tested in the study. In addition, it is observed that the findings obtained in the research are supported by the literature. In light of these findings, it is observed that cross-country skiing exercise may be an important tool for adolescent individuals in improving cognitive functions.

## Conclusion

Studies in the literature largely support the findings of a recent study that found that eight weeks of cross-country skiing exercise had a positive effect on the parameters of reaction, selective attention and academic achievement in adolescents. The study suggests that promoting exercise applications such as cross-country skiing, which requires complex and varied motoric skills, and making them a habit among adolescents, who are in the most critical stage of development, is essential for cognitive performance. The study recommends that parents’ guide their children towards exercise and sports applications, such as cross-country skiing, to improve cognitive and academic performance during adolescence. In addition, the research reveals the positive effects of cross-country skiing, one of the winter sports, on cognitive activities; This raises the question of whether winter sports or summer sports are more effective cognitive tools for adolescent athletes. When the branches commonly preferred by adolescent individuals or their families are examined today, it is observed that branches such as football, basketball, volleyball, handball, tennis and wrestling are at the forefront. It is thought that comparing adolescent individuals doing winter and summer sports in terms of cognitive activities in future studies will contribute to the field in terms of deepening the cognitive dimension of sports. It is thought that the findings resulting from such a comparison may provide a way for parents to direct their children to branches that will have a more positive cognitive impact. It is also thought that examining the positive effects of cross-country skiing on cognitive activities through similar studies may increase the tendency towards winter sports. It is thought that the research conducted in this context will contribute to the field in terms of making the positive effects of winter sports visible.

### Limitations

The sample group of the research was limited to adolescent individuals who had not done sports before. In addition, the cross-country skiing exercise program applied to the sample group was limited to 8 weeks. Cognitive parameters examined in the study; limited by reaction time, selective attention, and academic achievement.

## Data Availability

Data are available for research purposes upon reasonable request to the corresponding author.

## Abbreviations

R.T.: Reaction Time
R.C.R.: Reaction Correct Rate
C.R.: Correct Rate
